# The Obligations of the University to Medical Education*Read at the Joint Conference of the Council on Medical Education and Committee on Medical Legislation of the American Medical Association, Chicago, Feb. 28-March 2, 1910.

**Published:** 1910-06-15

**Authors:** Henry S. Pritchett

**Affiliations:** President of the Carnegie Foundation for the Advancement of Teaching; New York City


### THE OBLIGATIONS OF THE UNIVERSITY TO MEDICAL EDUCATION.*

HENRY S. PRITCHETT.
President of the Carnegie Foundation for the Advancement of Teaching
NEW YORK CITY

In an admirable paper before the Sixty-fifth Convocation of the University of Chicago, in December, 1907, Dr. William Henry Welch, professor of pathology in Johns Hopkins University, pointed out with clearness that the historical and right home of the medical school is the university, and that it should be an integral part of it, coordinate with other departments. In the discussion of this matter he called attention to the fact that no other type of medical school has existed on the continent of Europe other than that which was under the fostering care and which shared the ideals of the university. It was under similar ideals that the mecidal school in this country began, and Dr. Welch traced with admirable clearness the story of the transfer of medical education from the university to the proprietary medical school.

He called attention to the fact that the proprietary school in higher education is a unique institution in the history of education, one for which the United States of America is alone responsible. Dr. Welch traced then in the most interesting way the gradual recovery of medical education in this couutry during the last fifty years and showed that our medical education is today on the upgrade and that we are striving to remove it from the proprietary status and to bring it back once more into a close association with the university, where it may receive both the inspiration and the assistance which it must receive if it is to exist on a true basis.

Since the delivery of Dr. Welch's address two years ago, the Carnegie Foundation for the Advancement of Teaching,

*Read at the Joint Conference of the Council on Medical Education and Committee on Medical Legislation of the American Medical Association, Chicago, Feb. 28-March 2, 1910.

in conjunction with various medical bodies which are interested in this matter, has made a careful study of the present status of medical education in the United States and Canada. In the course of that examination every medical school in these two countries has been visited and studied. This includes the schools of every sect of medical practitioners. The material which this study has afforded is now being put into form for publication, and it is hoped that within two or three months it may be given to the public. To-day I shall endeavor to give some general results of that study and to give them in the light of the summary which I have just made from Dr. Welch, that is to say, in full recognition of the fact that the home of the medical school is in the university and that the gains which are to be had by the incorporation of the medical school as a part of the university are mutual, the university on the one hand offering a strong faculty and the medical school on the other hand conferring prestige and honor and scientific enthusiasm on the university. Such union must always be reciprocal if it is to be a true union; and there is no reason for the existence of the medical school in the university unless the university itself both confers and receives intellectual and educational help.

A brief statement of the situation which one actually finds in the United States and Canada to-day shows that there are in existence about one hundred and fifty medical schools. We have, in a word, more schools for the training of physicians and surgeons in the United States than are to be found on the whole continent of Europe. Of these, eighty-two are connected with actual colleges or universities. In making this count I leave out of consideration as university medical schools those under the control of such institutions as the University of Buffalo, the University of Maryland, and the like, in which the university is really fictitiuos. During the last ten years these one hundred and fifty medical schools have graduated on the average about five thousand physicians annually. How great an overproduction of physicians this is may be appreciated from a consideration of the following facts: From the experience in the older countries it seems clear that in large towns one physician for every 2,000 persons would, under present hygienic conditions, be sufficient. In more thinly settled regions one physician for every 1,000 inhabitants would suffice. In general, we may say that one physician for every 1,500 persons would be a fair ratio, and this is approximately that the older countries of Europe. The actual proportion in the United States at this moment is one physician for every 568 persons, which gives us twice as many physicians per thousand of population as England, four times as many as France, five times as many as Germany.

Consider it again from another point of view. The twelve southern states gained, in 1908, 358,837 persons. On the basis of one physician for every 1,500 inhabitants the section needed, in 1908, 238 more physicians. During the course of the same year there were 465 vacancies due to death. In view of the current overcrowding, these vacancies did not really need to be filled; but if we fill two vacancies with one new physician, 233 more graduates would have been needed; in all, 471 new men. As a matter of fact, 1,144 physiciams were graduated in southern schools and 78 more southerners in Baltimore and Philadelphia. The total would certainly come to 1,300 additional physicians, three times what was needed on a most liberal estimate. In the rest of the country the total gain in population was 975,000, to meet which 650 physicians would have been required. There were also 1,605 vacancies by death, to meet which would have required 800 more on the hypothesis just made. About 1,500 physicians would then amply have sufficed to fill the vacancies. As a matter of fact, 3,500 were produced. It seems clear that by the opearation of the great number of medical schools and the laxity of our laws we have for twenty years past graduated into the profession about three times as many men as are actually needed.

Before considering the duty of the university in the face of these conditions I wish to refer to one or two of the grounds on which this overproduction has been defended and which, from the material which has been gathered recently, are clearly shown to be untenable. In the inquiries which have been made concerning the reasons for the existence of various low-grade medical schools I have been met everywhere by the statement that it was the function of such a school to furnish doctors for rural regions. It was admitted that the training of these doctors was poor, but the constant assertion is made that the inhabitant of a small village or the resident on an isolated farm must accept this poor medical attendance or none at all. The investigation which we have made show conclusively that there is no truth whatsoever in this claim. It is perfectly clear from the statistics which have been brought together that a low basis of education does not force a distribution beyond a certain point. It may not be generally appreciated that the reverse of this is also true, namely, that a high basis of education does not prevent a wide distribution; that is to say, men graduated from good schools will go into all fields. The fact is that in the distribution of physicians many elements will count—the number available for distribution, home ties, individual tastes, professional opportunity, and financial consideration. The experience of foreign nations and our own shows that more expensively trained doctors will go into the country just as the poorly trained do, and it shows, on the other hand, that the ill-trained and poorly equipped men will in large measure congregate and get in each other's way. In proof of this statement it may be said that in Canada, whose laws for the admission to practice are far more advanced than ours, the well-trained graduates of McGill and of Toronto go in large numbers into the territories of the Northwest. In our own country the most expensive physicians whom we have yet graduated are Johns Hopkins men, and no one would propose to-day that the Johns Hopkins standards of admission and of facilities should be universally enforced. Nevertheless, these elaborately trained men have an astonishing disposition to scatter. They are to be found in thirty-two states and territories, and about a dozen have gone as missionaries to the Orient. One finds a Johns Hopkins man at Clayton, Ala., a town of 1,000; at Gorham, Colo., a town of 364; at Wells River, Vt., with 666; at Fairfax, Va., with 200; at Mazomanie, Wis., with 900, Western Reserve University is almost equally high in its entrance requirements, and one finds Western Reserve graduates in small towns of 700, 500 and 400 inhabitants. The argument that a cheap medical school must be maintained for the benefit of rural regions is absolutely without foundation.

A second argument which has been advanced wherever the low-grade medical school takes root is the time-worn argument on behalf of the poor boy. For example, in the city of Philadelphia, where medical facilities are offered on easy terms and under the most moderate conditions of entrance requirement, Temple University considered it necessary, for the sake of the poor boy, to maintain a night school of medicine. This argument, like the other, is based not on consideration for the poor boy, still less on consideration for the public, but on the desire to run a medical school. The truth is that the medical education which the poor boy thus gets costs him just as much as he would pay for something better. A four-year medical education in the Chicago, Baltimore, or Philadelphia schools on the ordinary basis costs in tuition fees and board $1,370. The same student can go to Ann Arbor for six years and save a few dollars; or if he wants a larger city, to the University of Minnesota at Minneapolis, for a little more. Now the absurdity is here: The low-grade medical school will tell you that their graduates can afford to go to small towns because they have not had an expensive education; well, then, so can the graduates of Ann Arbor and Minneapolis, for their education, though of higher grade, has been just as cheap. In a community where a fairly strong medical school is conducted it is possible to start a new school only by lowering standards, and it is this process which brings in the argument of the poor boy. The truth is that the poor boy has no right to enter the profession unless he is ready to make the sacrifices necessary for the right preparation. It is also true that in any large American community the poor boy will qualify himself rightly will find the way both to a fitting education and to an efficient medical training. The argument for the poor boy is in effect an argument for the poor medical school, and it has no reasonable foundation.

Taking the situation of medical education as it now stands, knowing, as we now know, how great an overproduction of physicians we have been making, the question to which I wish to bring attention is this: What is the duty of a university with respect to medical education under such circumstances? Granting, as one does freely, that the historic, natural home of the medical school is in a university, what is the duty of an institution toward the teaching of medicine in view of the present situation of medical education and of medical practice in America?

To answer this question fairly, one needs to consider with some detail what the concrete services are which a university can render to its medical school. In seeking to answer that question, one will keep in mind the fact that, while medicine has always been a learned profession, it is to-day true that there is a vast difference between the scientifically trained physician and the able practitioner of earlier days. Within twenty-five years the development of certain fundamental sciences has been so great that the practice of medicine has come to be almost the application of these sciences—or at least this much may be said: the practice must start and rest as it did not in the preceding days on these fundamental sciences—pathology, anatomy, physiology, physiologic chemistry, bacteriology, and the like. This means that to-day we seek to train in our medical schools, as always, the pratitioner of medicine, but the pratitioner of medicine to-day is a scientifically trained man as he never was before. It does not mean that the analysis of the chemical laboratory or of the biologic laboratory will ever replace the skill of the clinician. It will always be true, so far as we can see, that in the treatment of diseases clinical skill and experience will be the largest factor in the physician's equipment, but it does mean that clinical skill to-day is supplemented with aids which the generation before did not know, and that the pratitioner who is trained in a school which is unable to give these fundamental facilities is practicing the profession of a generation ago.

In view of the fact that the clinician is still the guiding spirit in medical education, it will always remain true that a medical school must be made up of school, hospital, and dispensary, operated as a single working plant.

Taking into account the present status, therefore, of medical education and taking into account also the standards of training which modern practice demands, it seems to me that the concrete services which the university can render to its medical department may be grouped under three heads:

1. The definition and maintenance of entrance standards.

2. The inspiration of the medical school with an appreciation of productive ideals both in education and in science.

3. The financial support of the medical department exactly on the same basis as other departments are supported.

To put the matter in a single sentence, a university which understands the present situation in medicine, which appreciates the ideals of modern medicine and which appreciates as well its own duties and responsibilities, will make itself responsible for the standard of admission to its medical school, for the ideals which govern it, and for the financial support which is to maintain it.

What is a reasonable basis, what is a reasonable standard to exact as the basis of a medical education? It is generally conceded that a scientific training in physics, chemistry and biology constitutes an almost indispensable preliminary qualification for the study of medicine. As our present educational system stands, it is practically impossible for the student to obtain this equipment short of two years of college study, which would mean his entrance into the medical school at approximately twenty years of age.

When one comes to consider the enforcement of such a standard, however, it is clear that one must take into account the status of educational facilities in the various sections of the country. No standard can be enforced for which the schools and colleges of the region cannot reasonably well fit students. Our present educational facilities in the eastern and middle states and in the western states are already so considerable, they are so rapidly increasing, and they respond so quickly to pressure of the right kind that there is no question that the requisite number of physicians could be obtained from these sections even with the enforcement of such a standard as that indicated. The only practical difficulty that stands in the way is that the enforcement of such a standard would involve the death of most of the medical schools now existing in these regions.

In the South matters are very different. Both the legislative conditions and the educational facilities in that region are such that the standard just proposed, which is clearly a fair one for the profession itself, could not possibly at this moment be suddenly applied. This, however, is no excuse whatsoever for the almost utter absence of standards which is characteristic of that section. Only three complete institutions in the twelve Southern states have anything that can be definitely called a standard of admission for their medical schools. These are Tulane, the University of Texas, and the University of Virginia. Inasmuch as standards in medical education are necessarily tied up with the general educational system, I venture to suggest that the feasible standard in the Southern states at this moment for entrance into the medical schools is the same as that for entrance to the academic department, that is to say, high school preparation. This is the level which the general school situation of the different states either reaches at this moment or will reach in the near future. From this point progress may be gradual, but it will be inevitable. I venture to point out also that the enforcement of even this standard in the South will bring about the extinction of most existing superfluous schools.

One more thing I venture to add concerning the matter of standards of admission to the medical school, a statement clearly applicable, however, to the American university throughout all its courses, and that is that no increase of standards is helpful which does not mean actual enforcement. The conscientious and just enforcement of the four-year high-school standard throughout the whole country would alone go far toward clearing the situation; but in any case a university which undertakes to maitain a medical school ought clearly to face the duty of maitaining in its medical school standards fully abreast of the educational status of its region. If an institution is not willing to do this, then it ought not to meddle with medical education at all. The college or the university which maintains a decent standard of admission for its college and a far lower one for admission to its medical school is frankly disregarding its own duty to medical education and is seeking, as a rule, to support a medical school out of fees in order to have a certain institutional completeness. The day has gone by in the United States when an institution calling itself a university can do this without being recognized as shirking the responsibilities of a true university.

There are in the United States a few institutions of high grade which require college graduation for admission to their medical schools. We may rejoice to have this standard set up in a few of our best schools. I do not believe, however, that it will be generally adopted, and largely for the reason that, however necessary it is to give the medical practitioner the advantage of a liberal education, it is also important for him to get into the profession before he is to old.

With regard to the second specific service of the university to the medical school, namely, as to its influence on the pedagogic methods and the scientific ideals, I venture at this point merely to call attention to the very general neglect of this function of the university in most American institutions. The American medical school, even when it has been incorporated into the university itself, has remained in many cases an *imperium in imperio.* There has been, as a rule, little commerce between the professors of medicine and the professors of other faculties of the university. This has been due in part to the fact that the professor of the medical school has been in the past a practicing physician coming into the school for only brief lecture hours and having little touch with teaching or with the members of the other faculties. In the best schools to-day, where not only the professors of physiology, pathology, chemistry, anatomy, and biology are men giving their whole time to the work, but where also the professors of medicine, of surgery. of pediatrics, are coming to be placed on an explicit teaching basis, the opportunity for the development of a true university spirit in the medical school is beginning to appear. This ought to result in the spread of the scientific spirit through the medical school; and while it will always remain true that the chief function of the medical school is to train the medical practitioner, it will also be true that the spread of university ideals will mean that more men will take up the investigation of medical problems from the scientific standpoint and that our medical schools, so organized, will lend themselves in increasing measure to the development of the scientific side of medicine and the investigation of its problems.

I venture here to allude to one feature of our American medical school organization which, from the standpoint of one long associated with an institution of learning has struck my attention forcibly, and that is the project for a divided medical school. It seems clear to me that such a school has distinct disadvantages and that the very division gives up in large measure the opportunity of the university to create in the medical school university ideals. It is difficult to understand, for example, how a medical school in which the clinical school is two hundred miles away from the scientific laboratories can receive any great inspiration from the men who work in these laboratories. Still more difficult must it be for the clinician, whose spirit ought to dominate the whole institution, to keep the laboratory methods and the laboratory men in touch with clinical aspirations and clinical needs. It may be that the divided medical school will succeed, but it seems that it must succeed against much greater obstacles than the school which is organically one. I cannot help wondering, for example, how it would affect the pedagogic and professional ideals of an engineering school if the first two years of it were given in one place and the last two years in a place two hundred miles away. My impression is that there would be two separate schools with very little more reaction, the one on the other, than exists between any other two schools so situated.

With respect to the third service which the university ought to render to its medical department, namely, that of an adequate financial support, I wish to speak with some degree of definiteness. This obligation is one which few colleges or universities in our country have frankly faced. In more than one report of university presidents during the past year I have read the boast that the medical school has not cost the university a cent! Colleges and universities have taken over medical schools in distant places with no expectation whatsoever of furnishing any support, and generally throughout the relations which have existed between colleges and universities on the one side and medical schools on the other there has been a most remarkable absence of any sense of financial obligation on the part of the university. I am convinced that this action has been due in the main to a lack of knowledge on the part of college and university trustees and officers. They have come to look on medical education as self-supporting; they desire a medical school in order that their institution may seem complete. The overproduction of physicians is a matter with which they are not acquainted, and the college and university have gone ahead to take over a medical school with absolutely no thought of its support—a thing which they would never do with a school of applied science. Just why it should be believed that a medical school could be sustained by moderate fees, while an engineering school or a college of arts needs a large and strong endowment, is a matter which I have found it difficult to understand. Furthermore, when an engineering school undertakes to sustain itself in the main from fees, it exacts a fee somewhere comparable with the cost of the education. Thus the Institute of Technology at Boston, which is sustained in the main from its fees, charges a tuition fee of $250 a year and laboratory fees in addition, which amount in some cases to $50 more. Such a fee for a medical department would be thought impossible.

What does it cost to run an adequate, all-round medical school? By an all-round medical school I mean one in which all departments are fairly strong. It will be found that some schools of fairly high reputation have maintained themselves by making a single department strong and leaving all the rest weak.

The investigations of the Carnegie Foundation prove that it costs easily $10,000 a year to conduct any one scientific department of the first two years of medical education in a satisfactory fashion for classes of fair size. This means the teaching of a comparatively small number of students and makes relatively slight provision for any scientific activity on the part of the teachers. A number of institutions that are economically conducted spend $15,000 or more a year on a single subject like pathology or physiology, including a fair provision for investigation, and these are not institutions which one would think of as most liberally endowed. A medical school, therefore, which is not in a position to spend $50,000 or $60,000 a year on the work of its first two years has omitted some part of its legitimate duty. It will be difficult for any complete medical school to do its fair duty by a moderate body of students with an income of less than $100,000 a year. Furthermore, this cost will increase rather than diminish, for the reason that with the improvement in the training of the first two years correspondingly improved clinical teaching will be required. It has been one of the anomalies of this whole development to note that many medical schools have acted on the idea that standards of admission could be raised without further provision for an improvement in the teaching facilities and in the teaching itself. It requires only a year's experience, however, for such a school to appreciate the fact that young men who have had two years of college training will not be satisfied with the kind of teaching nor with the facilities which will be accepted by boys fresh from the high school. The college trained man becomes restless under the physician who drops in to lecture and spends half the time in telling stories from his medical practice, and he is not at all willing to sit under a teacher whose knowledge of his subject is evidently gathered from the text-book before him. The raising of standards of admission means an important increase in expense on account of the quality of the instruction. An institution which undertakes the teaching of medicine on a basis much lower than that which I have indicated is not likely to serve either the cause of education or the cause of medical practice, and an institution which finds itself facing the question of the creation of a medical school or of the adoption of one should fairly also face the question whether it can get the means necessary for its support. It goes without saying that no university is justified in entering into any arrangement for a medical school which rests on a contractual relation. Unless the university can control, inspire, and support the medical school, it has no right to it.

Few institutions in the United States or Canada have lived up to their obligations to their medical departments in all three of the essential respects to which I have referred. Some have not even appreciated any one of them. As I have before remarked, eighty-two of the one hundred and fifty medical schools of the country are connected with actual colleges and universities. Of these, perhaps one-half fulfil all three of these obligations in a reasonable degree, in the sense that the universities fix and enforce definite standards for entrance, secure the same sort of teaching and scientific activity in the medical department as in others, and finally, make themselves responsible for funds that go far beyond tuition fees received by the medical department. Of these institutions it may be fairly said that they are alive to and meet the responsibilities which they have undertaken in the matter of medical education.

There is in the next place a group of medical schools legally and organically parts of a college or university, but neglected by the institutions of which they are a part in one or more of the three services to which I have referred. It is difficult to say what these departments gain by their university connection. They receive no support, they get no oversight, they do not breathe the university air, nor receive any inspiration from university ideals. For example, the medical departments of Vanderbilt, Northwestern, Tufts, Bowdoin, administer in their own way the entrance requirements which the university sets up, and in none of these cases are the entrance requirements strictly enforced. In the matter of support, the university is often a harsh creditor to its medical department, sometimes not even lending it a dollar and at other times exacting, as in the case of Vanderbilt and Tufts, principal and interest for every advance made out of the general funds. As these institutions, organic university departments though they be, live on their fees or less than their total fee income, it is clear that the ideals of the university do not materially affect them. They are for the most part routine teaching schools, in no true sense university schools. Some of these organic medical departments belong to universities which can hardly hope by anything short of a miracle to do anything for medical education. For example, Drake University, with total productive funds of $290,000 has an organic medical department. The University of Alabama, with a total state appropriation for buildings and maintenance of $71,000 a year, has such a department. So have the University of Mississippi with similar appropriations of $110,000, the University of North Carolina with an annual appropriation of $70,000, the University of Tennessee with an annual maintenance appropriation of $65,000. It is perfectly clear that the problem of medical education is begun rather than solved when a university incorporates into its organization a medical school.

Twenty-seven of the remaining colleges and universities of the United States and Canada have nominal or affiliated medical departments which they not only do not support, but do not control. The state universities of Arkansas, Georgia, Illinois, and Oregon are in this position. Among endowed institutions that lend their names to proprietary medical schools for which they can hope to do nothing and which they do not control so long as they are in this situation are the University of Denver, Washburn College, Cotner University, Ohio Wesleyan University, Epworth University, Baylor University, and Dalhousie University. The budgets of some of these institutions are absurdly small in comparison with the cost of a decent medical school. Among them are some which are capable of leading respectable lives as colleges, but are little less than impossible as universities—for example, Union University, New York, which is the name given to the superficial combination of Union College with weak schools of law and medicine in Albany.

In a word, an inspection of the eighty-two medical colleges in the United States and Canada which are associated with colleges and universities shows that the relation is in many cases a superficial one. The college or the university has not in these instances approached the problem of medical education with serious purpose or with any fair sense of its responsibilities. It has been willing to give its name to a medical school, no matter how far away it was from university ideals and scientific traditions. In truth, the colleges are in no position to throw stones at proprietary medical schools.

If medical education in the United States is within the next decade to be raised to a fair standard, it is clear that the majority of the one hundred and fifty medical schools now existing ought to die. The country has no need whatsoever for even one-third of that number. Less than fifty decently equipped schools would more than supply all the needs of the country for a century to come.

The question whether we shall be able to bring public opinion to some fair understanding of this question and to secure such legislation as will provide a right scrutiny for candidates for the profession rests in considerable measure on two factors, which are moral rather than educational—educational patriotism on the part of the colleges and medical patriotism on the part of the physician.

By educational patriotism I mean this: A college has a duty greater than the mere ambition for a large student body or for institutional completeness, namely, the duty of loyalty to certain standards of common honesty, of intellectual sincerity, and of workmanlike skill, from which it cannot afford to depart. The combination of these qualities means a loyalty to the ideals of education itself, a patriotism larger than the intersts of the colleges and taking hold on the very ideals of civilization. A university with such educational patriotism will not take on a medical school unless it can discharge its fullv duty by medical education; or if, in the days of ignorance which was once winked at, it became entangled in such an alliance, it will frankly and courageously go to work to get rid of a situation which is no longer tenable, for the day has come when all colleges everywhere are called on to repent. The flirtations between universities and medical schools ought now to cease and those institutions which have become involved in this sort of relation ought either to marry or get a divorce. The college which develops educational patrotism will be ready to do its duty toward the general system of education and toward the interests of professional education as well, and the question whether the inefficient medical school is to disappear or not will in large measure depend on the growth of a true university patriotism which looks on educational sincerity and thoroughness as something greater than institutional completeness.

By professional patriotism among medical men I mean that sort of regard for the honor of the profession and that sense of responsibility for its efficiency which will enable a member of that profession to rise superior to the consideration of personal advantage or of social cliques. As Bacon truly wrote: "Every man owes a duty to his profession," and there is no professional man whose obligation is more clear than that of the modern physician and surgeon. He is the outcome of the culminating forces of civilization, and he owes his very existence to the progress of these forces. For a hundred years past medical education has been a perquisite of the American medical profession, a thing which has been used both as a direct means of revenue and as a source for wide circle of patients. When a particular group of physicians in a given city have been left out of existing medical schools they have promptly got together and made a medical school of their own. The day for the proprietary medical school has gone by. How long they can still survive will depend in large measure on the spread among American physicians and surgeons of a medical patriotism which will enable them to accept the new order of things and to come to the aid of the reputable universities in the support of medical schools where ideals of high professional honor and efficiency may be placed before the eyes of those who come to study in them.

I commend to the thoughtful consideration of medical men everywhere—those with and those without school connection—the wise, self-denying action of the physicians of Seattle, who, recently stirred by the honor of a projected medical school on local lines, went on record as a body against any attempt to organize a medical school until the state university of Washington could undertake the task with credit to itself, to the medical profession, and to the people of the state.—*A. M. A. Journal*, April 2, 1910.

